# Syphilis—Is It Ever a Cause of Phthisis?

**Published:** 1876-08

**Authors:** 


SYPHILIS—IS IT EVER A CAUSE OF PHTHISIS?—Dr. N. W. Thoreson (*Norsk. Mag. for Laegeridenskaben—N. Y. Med. Jour.*, September, 1875) gives us the result of an able investigation of this subject. The history of three hundred and fifty syphilitic parents is given, as well as that of their children. It was often found that a syphilitic mother gave birth to a healthy child, which was nursed by her and remained healthy, and if the mother was not treated the next child might have marked hereditary syphilis, or present other signs of ill health. In several instances several healthy children were successively born of such a mother, who then gave birth to syphilitic children. Others had syphilitic children, and then gave birth to perfectly healthy ones. In no case, however, could the origin of phthisis be traced to syphilis in the parents. It was constantly found that where the children had tubercles, there was a history of this disease in their ancestors. Of the whole number of syphilitic individuals examined, there were only sixteen who had tuberculosis. In all of these there was a family history of tuberculosis. In nine tuberculous individuals who became syphilitic, the course of the latter was very disastrous. The condition of those twelve tuberculous persons who had not had tuberculous deposits was carefully watched, and though most of them had severe syphilitic accidents, none of them had any chest affection, or any other manifestation of tuberculosis. The auther concludes that syphilis has no casual relation whatever to tuberculosis, though the latter discrasia renders the prognosis of syphilis bad, just as any other depressing condition would.—*Detroit Review of Medicine.*

